# Defining Multimorbidity: From English to Portuguese Using a Delphi Technique

**DOI:** 10.1155/2015/965025

**Published:** 2015-11-23

**Authors:** Filipe Prazeres, Luiz Miguel Santiago, José Augusto Simões

**Affiliations:** ^1^Faculdade de Ciências da Saúde, Universidade da Beira Interior, 6200-506 Covilhã, Portugal; ^2^Centro de Saúde de Aveiro, 3810-042 Aveiro, Portugal; ^3^USF Topázio, 3020-171 Coimbra, Portugal; ^4^USF Marquês de Marialva, 3060-123 Cantanhede, Portugal

## Abstract

*Objective*. To translate the European General Practice Research Network multimorbidity definition according to Portuguese cultural and linguistic features.* Methods*. Similar to the process completed in several other European countries, a forward and backward translation of the English multimorbidity definition using the Delphi technique was performed in Portugal.* Results*. Twenty-three general practitioners (GPs)—14 males and 9 females—agreed to form the Portuguese expert panel for the Delphi process (59% acceptance rate). The Portuguese definition of multimorbidity was achieved after two Delphi rounds with a mean (SD) consensus score for final round of 8.43/9 (0.73).* Conclusion*. With this paper the definition of multimorbidity is now available in a new language—Portuguese. Its availability in the local language will raise Portuguese GPs' awareness about multimorbidity and allow future national and international research. The operationalization of the definition will allow an easier identification of patients with multimorbidity.

## 1. Introduction

Clinicians working in the primary health care context, namely, family physicians and general practitioners (GPs), deal with the broad spectrum of conditions affecting each individual seeking a medical consultation. In this setting, most of the time it is not possible to pinpoint an index disease, nor is it useful for the patient's care [1]. Therein lies the main difference between comorbidity and multimorbidity; the former always involves the presence of an index disease [2]. Thus, the majority of GP visits comprise individuals with multimorbidity [3]. The most frequent measure of multimorbidity is the presence of 2 or more chronic diseases in the same person [4]. Although this is a useful operational definition, the construct of multimorbidity is still difficult to define in clinical terms [5]. Recently, after a systematic literature review, the European General Practice Research Network published a comprehensive definition which states that “multimorbidity is defined as any combination of chronic disease with at least one other disease (acute or chronic) or biopsychosocial factor (associated or not) or somatic risk factor. Any biopsychosocial factor, any somatic risk factor, the social network, the burden of diseases, the health care consumption, and the patient's coping strategies may function as modifiers (of the effects of multimorbidity). Multimorbidity may modify the health outcomes and lead to an increased disability or a decreased quality of life or frailty” [6]. This definition aims to be especially useful in long term care and in family medicine settings [6] and at the same time to be valid for future collaborative research [7]. For this last purpose, it has been translated into ten European languages [7]. The Portuguese language was not one of them.

In Portugal, the 40-year history of family medicine led to the recognition of its importance in the country's health care delivery [8]. Multimorbidity is present in around 70% of the adult patients attending primary care in Portugal [9], and this high prevalence will produce significant difficulties in the provision of medical care. Using a definition of multimorbidity in the country's own language will standardize the identification of multimorbid patients while simultaneously enabling future collaborative projects as well as addressing more effectively this overwhelming medical problem.

It is expected that this definition will have a broad suitability to other Portuguese language settings and countries. The British Council's report “Languages for the Future” [10] identifies Portuguese as one of the ten languages most vital to UK over the next 20 years. With approximately 203 million speakers, Portuguese is the sixth most spoken language in the world [10], the third most spoken language in the Western Hemisphere, and the first most spoken language in the Southern Hemisphere [11].

In this study, the authors aimed to translate the English multimorbidity definition according to Portuguese cultural and linguistic features using a forward-backward translation by a Delphi technique.

## 2. Materials and Methods

Similar to the process completed in Bosnia, Bulgaria, Croatia, France, Germany, Greece, Italy, Poland, and Spain [7], a forward and backward translation of the English multimorbidity definition [6] using the Delphi technique was performed in Portugal. This technique is easily adapted to reach a consensus in a variety of issues [12], including medical research [13].

The first phase involved translating the definition from English to Portuguese (forward translation). This was done by a team of one official translator and one physician; both were native Portuguese speakers.

In the next phase the Delphi process was implemented. Aiming at a sample size between 10 to 30 national expert GPs as recommended by the European General Practice Research Network [7], 39 possible participants were individually contacted by email to receive the original English multimorbidity definition and its translation into Portuguese. GPs were selected on the basis of having a Portuguese nationality, being fluent in English (understanding/speaking/writing), being involved in clinical practice, in research, and/or in teaching activities, and having the willingness to dedicate the time to this method of discussion. The expert panel was requested to rate their level of agreement with the Portuguese translation on a Likert-type scale ranging from 1 = “absolutely no agreement” to 9 = “full agreement.” If a rating less than 7 was given it was mandatory to justify the reasons for that evaluation. Consensus was defined as at least 70% of the GPs rating 7 or above the Portuguese definition. If a consensus was not reached in the first round, the expert panel's remarks were compiled into a unified translation, and a subsequent round of assessment was followed in the same way as for the first one. This process was repeated until a consensual translation was found. The participating GPs' characteristics (gender, age, years of practice, number of English publications, and number of other publications) were collected by a self-administered questionnaire conducted through email.

When a consensual Portuguese translation was reached it was submitted to a Portuguese linguist from the University of Coimbra (Portugal) for validation.

The final phase involved translating the consensual definition in Portuguese to English (backward blind translation). This was done by a team of one official translator (native English speaker) and one physician. They had no previous knowledge of the original definition. Subsequently, the authors of the study compared the back-translated version with its original version for linguistic congruence and cultural relevancy.

As no patient was involved in the study, no formal ethics approval was necessary. Consent was inferred by participants' completion of the survey.

A descriptive analysis was performed and both Fisher's exact test and Student's *t*-test were used to compare the current study's expert panel with the panel of the previous translations. *P* values <0.05 were considered statistically significant.

## 3. Results

Twenty-three GPs (14 males and 9 females) agreed to form the Portuguese expert panel for the Delphi process (59% acceptance rate). All members of the expert panel satisfied the inclusion criteria. The profile of the Portuguese GPs did not differ significantly from that of the previous translations [7] (Table 1).

The Portuguese definition of multimorbidity was achieved after two Delphi rounds with a mean (SD) consensus score for final round of 8.43 (0.73). Only one expert rated the forward translation below 7 (95.7% approval rate). The expert panel produced 43 comments in total. The terms which originated remarks were “burden of disease” and “health outcomes.” Minor grammatical annotations were frequently suggested, recorded, and incorporated into the definition.

Table 2 shows the final consensual Portuguese definition of multimorbidity and the backward translation as accepted by the authors of this study. No changes were found in comparison with the original English definition.

## 4. Discussion

With the current study the translation of the English multimorbidity definition into Portuguese was achieved.

No universal guidelines exist on how to apply the Delphi technique [14]. Some authors have even stated that the advantages and disadvantages of this method are equally weighted [12]. Nonetheless, with methodological precision and research rigour the Delphi technique can be properly and efficiently used [14]. In the current study, the successful methodology employed in previous translations was adopted.

The Portuguese translation was the end result of the reviews of an expert panel of practicing GPs that verified that the terms expressed in the definition complied with the ones in use in Portugal. The Portuguese panel had similar characteristics to the average of the panels of the previous translations [7]. This ratifies the thorough selection process used to choose the GP experts in this study. The challenged terms were the same as in the other countries' translations; this may be explained by the fact that those expressions are less commonly used on a daily basis. In the second round this was overcome and the backward translation did not reveal any changes in comparison with the original English definition.

## 5. Conclusion

With this paper the definition of multimorbidity is now available in a new language—Portuguese. Its availability in the local language will raise Portuguese GPs' awareness about multimorbidity and allow future national and international research. The operationalization of the definition will allow an easier identification of patients with multimorbidity.

## Figures and Tables

**Table 1:** Characteristics of the expert panel.

	Portuguese translation (*n* = 23)	Global average of previous translations [7] (*n* = 229)	*P* value
Males, %	60.87	50.69	0.51^*∗*^
Mean (SD) age, years	45.78 (12.82)	48.26	0.36^†^
Mean (SD) years of practice	18.09 (13.28)	18.82	0.79^†^
Mean (SD) number of English publications	6.13 (7.12)	5.91	0.88^†^
Mean (SD) number of other publications	15.09 (15.24)	20.45	0.11^†^

^*∗*^Fisher's exact test.

^†^Student's *t*-test.

**Table 2:** Portuguese final translation and the backward translation.

Portuguese final version	Portuguese final version translated into English
A multimorbilidade é definida como qualquer combinação de uma doença crónica com pelo menos uma outra doença (aguda ou crónica), ou com um fator biopsicossocial (associado ou não), ou com um fator de risco somático.	Multimorbidity is defined as any combination of chronic disease with at least one other disease (acute or chronic) or biopsychosocial factor (associated or not) or somatic risk factor.
Qualquer fator biopsicossocial, qualquer fator de risco somático, a rede social, a carga das doenças, o consumo de cuidados de saúde e as estratégias de adaptação do doente podem funcionar como modificadores (dos efeitos da multimorbilidade).	Any biopsychosocial factor, any somatic risk factor, the social network, the burden of diseases, the health care consumption, and the patient's coping strategies may function as modifiers (of the effects of multimorbidity).
A multimorbilidade pode modificar os resultados em saúde e levar a um aumento da incapacidade, à diminuição da qualidade de vida ou à fragilidade.	Multimorbidity may modify the health outcomes and lead to an increased disability or a decreased quality of life or frailty.
